# Comparing Diagnostic Accuracy of Clinical Professionals and Large Language Models: Systematic Review and Meta-Analysis

**DOI:** 10.2196/64963

**Published:** 2025-04-25

**Authors:** Guxue Shan, Xiaonan Chen, Chen Wang, Li Liu, Yuanjing Gu, Huiping Jiang, Tingqi Shi

**Affiliations:** 1 Nanjing Drum Tower Hospital Clinical College of Nanjing University of Chinese Medicine, Nanjing, China; 2 Jiangsu Province Hospital of Chinese Medicine, Affiliated Hospital of Nanjing University of Chinese Medicine, Nanjing, China; 3 Department of Emergency, Nanjing Drum Tower Hospital, Nanjing, China; 4 Department of Nursing, Nanjing Drum Tower Hospital, Nanjing, China; 5 Department of Quality Management, Nanjing Drum Tower Hospital, Affiliated Hospital of Medical School, Nanjing University, 321 Zhongshan Road, Gulou District, Nanjing, 210008, China, 86 1-391-299-6998

**Keywords:** machine learning, ML, artificial intelligence, AI, large language model, LLM, natural language processing, algorithm, model, analytics, NLP, deep learning, clinical diagnosis, diagnosis, diagnostic accuracy, accuracy, systematic review

## Abstract

**Background:**

With the rapid development of artificial intelligence (AI) technology, especially generative AI, large language models (LLMs) have shown great potential in the medical field. Through massive medical data training, it can understand complex medical texts and can quickly analyze medical records and provide health counseling and diagnostic advice directly, especially in rare diseases. However, no study has yet compared and extensively discussed the diagnostic performance of LLMs with that of physicians.

**Objective:**

This study systematically reviewed the accuracy of LLMs in clinical diagnosis and provided reference for further clinical application.

**Methods:**

We conducted searches in CNKI (China National Knowledge Infrastructure), VIP Database, SinoMed, PubMed, Web of Science, Embase, and CINAHL (Cumulative Index to Nursing and Allied Health Literature) from January 1, 2017, to the present. A total of 2 reviewers independently screened the literature and extracted relevant information. The risk of bias was assessed using the Prediction Model Risk of Bias Assessment Tool (PROBAST), which evaluates both the risk of bias and the applicability of included studies.

**Results:**

A total of 30 studies involving 19 LLMs and a total of 4762 cases were included. The quality assessment indicated a high risk of bias in the majority of studies, primary cause is known case diagnosis. For the optimal model, the accuracy of the primary diagnosis ranged from 25% to 97.8%, while the triage accuracy ranged from 66.5% to 98%.

**Conclusions:**

LLMs have demonstrated considerable diagnostic capabilities and significant potential for application across various clinical cases. Although their accuracy still falls short of that of clinical professionals, if used cautiously, they have the potential to become one of the best intelligent assistants in the field of human health care.

## Introduction

The Google Brain research team has consistently aimed to push the boundaries of recurrent language models and encoder-decoder architectures. In 2017, Vaswani et al [1] introduced a novel and simple network architecture known as the Transformer. This architecture uses a new mechanism called “self-attention,” leading to significant advancements in the development and training of large language models (LLMs). These models possess advanced capabilities beyond extraction or summarization tasks and include natural language generation. Although there is no official definition of LLM, based on the literature [2,3], we define LLM as a model with over a billion parameters, designed for typical artificial intelligence (AI) applications.

Accurate clinical diagnosis is essential for patient treatment outcomes and survival rates. However, even when health care professionals gather extensive information and conduct numerous observations and tests, absolute diagnostic accuracy cannot be guaranteed. Minimizing diagnostic uncertainty and making the most appropriate treatment decisions remain persistent clinical challenges [4,5]. As of May 2024, the US Food and Drug Administration has approved 882 medical devices that use AI or machine learning assistance. By June 2024, the National Medical Products Administration of China has approved 17 AI-assisted diagnostic devices. In the era of big data in health care, the integration of AI with clinical decision support is a developing trend [6]. Numerous experts and scholars have explored the application of specialized AI and software tools in clinical diagnosis, yet there is limited knowledge about the performance of LLMs in this context. Therefore, this study aims to comprehensively evaluate the performance and accuracy of LLMs in clinical diagnosis, providing references for their clinical application.

## Methods

### Overview

This systematic review was conducted following the Preferred Reporting Items for Systematic Reviews and Meta-Analysis of Diagnostic Test Accuracy Studies (PRISMA-DTA) statement [7]. Specific details can be found in Checklist 1.

### Data Sources

A computer-assisted literature search of PubMed, Web of Science, Embase, CINAHL (Cumulative Index to Nursing and Allied Health Literature), CNKI (China National Knowledge Infrastructure), VIP, and SinoMed databases was performed from January 1, 2017, to the present. Search terms included controlled terms (MeSH [Medical Subject Heading] in PubMed and Emtree in Embase) and free-text terms. The following terms were used (including synonyms and closely related words) as index terms or free-text words: “large language model,” “medicine,” “diagnosis,” and “accuracy.” A search filter was applied to limit the results to humans. Only peer-reviewed cross-sectional studies and cohort studies were included. Multimedia Appendix 1 provides more details of the search strategy and study selection.

### Selection Criteria

This review included studies meeting the following criteria: (1) investigated the application of LLMs in the initial diagnosis of human cases, (2) published between January 1, 2017, and the date of the search, (3) study type was either cross-sectional or cohort, (4) a primary source, and (5) written in English or Chinese.

An article was excluded if it (1) was a nonprimary source such as theses, conference papers, etc, (2) did not compare the diagnostic accuracy of clinical professionals in relevant departments with that of LLMs, (3) did not specify the type or scale of the LLM used for diagnosis, (4) did not have LLM independently conduct clinical case diagnoses, (5) was a duplicate publication, and (6) did not provide complete data or the full text could not be obtained.

### Data Selection and Extraction

A total of 2 reviewers (GS and XC) independently reviewed the full texts of the eligible articles and extracted data. Any disagreements between the reviewers were discussed until a consensus was reached. The detailed characteristics extracted from each included study were: the first author and publication year, the country where the research was conducted, the study type, the study population, the source of cases, sample size, the LLMs used, control groups, and outcome measures.

### Quality of Evidence and Risk of Bias

Due to the significant heterogeneity often present in the design and implementation of diagnostic accuracy studies, it is crucial to carefully assess the quality of the included studies. The Prediction Model Risk of Bias Assessment Tool (PROBAST) was used to evaluate the risk of bias and applicability of all included studies [8]. PROBAST assesses risk of bias across 4 domains: study participants, predictors, outcomes, and statistical analysis, while applicability is evaluated through the first 3 domains.

Given the complex structure and vast number of parameters in LLMs, they can be considered a “black box” to some extent, meaning that their internal workings and decision-making processes may not be entirely transparent or easily understood by humans [6]. Consequently, during the quality assessment, certain signal issues were excluded as they were unrelated to generative AI models [9].

## Results

### Selection of Studies

**Figure 1.**
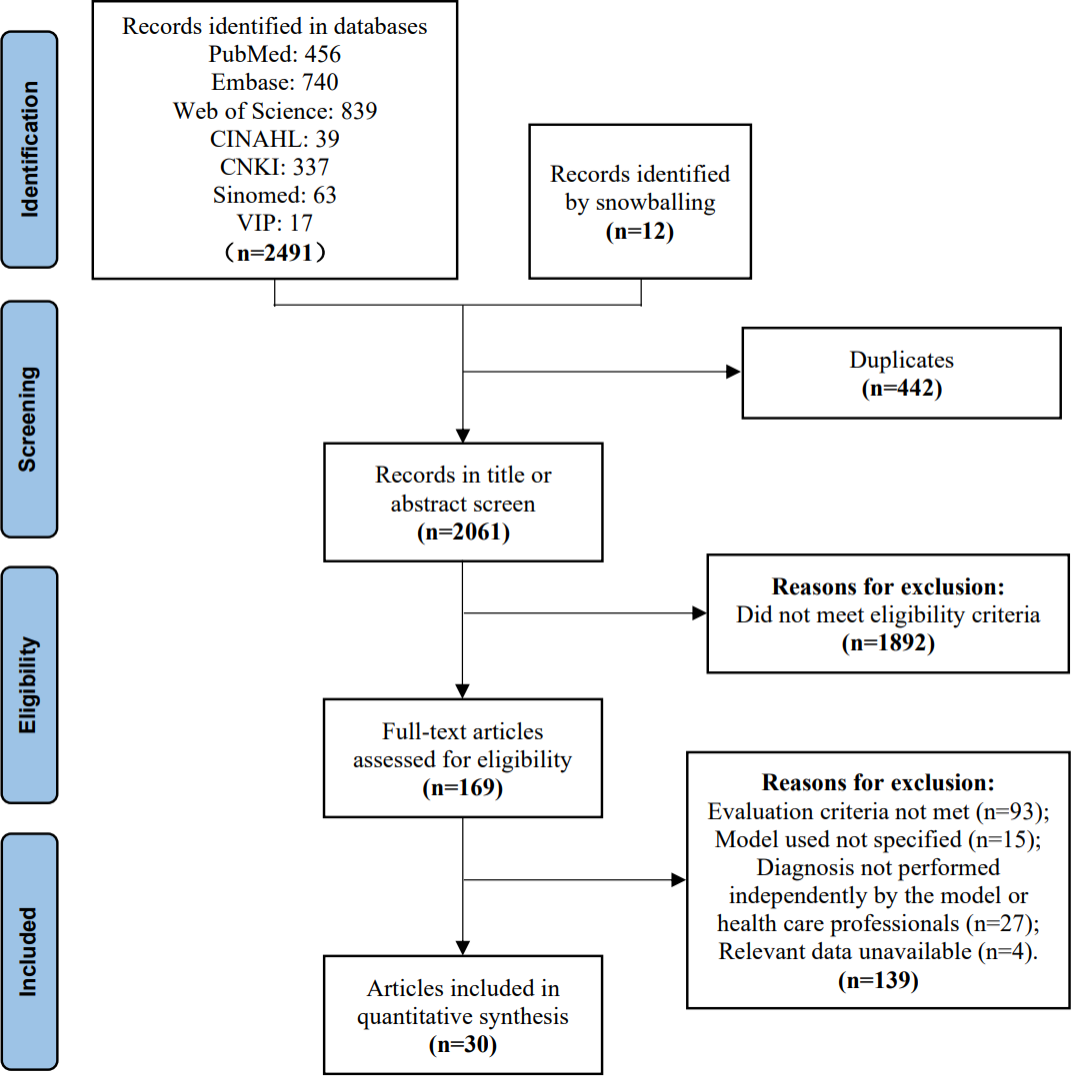
Flow diagram. Papers identified in databases, title or abstract screened, read full text, and included in the synthesis. Reasons for exclusion are listed. CINAHL: Cumulative Index to Nursing and Allied Health Literature; CNKI: China National Knowledge Infrastructure.

A total of 2491 studies were found in the databases by 2 researchers independently following the predefined search strategies and data collection methods. An additional 12 articles were identified through reference tracing, bringing the total number of studies screened to 2503. Among these, 169 studies were read in full, resulting in 30 studies that met the inclusion criteria for synthesis. Reasons for exclusion at this stage were recorded and can be found in the flow diagram (see Figure 1).

### Studies Characteristics

The 30 included studies [10-39] were concentrated within the past 3 years, with 12 published in 2023, 16 in 2024, and 2 in 2025. These studies cover a wide range of countries, primarily from Japan, the United States, and China. A total of 4762 cases were analyzed, involving 19 LLMs. The studies predominantly focused on GPT-3.5 (n=14) and GPT-4 (n=20) versions (OpenAI), extensively applied in assessing clinical diagnostic accuracy. In contrast, fewer studies addressed Google Bard (n=3), Bing (n=3), GPT-4o (n=2), and GPT-4V (n=2). The case diagnoses encompassed various fields, including ophthalmology (n=9), internal medicine (n=6), emergency medicine (n=3), and general medicine (n=3), among others. The control groups included at least 193 clinical professionals, ranging from resident doctors to medical experts with over 30 years of clinical experience, to compare their diagnostic capabilities with those of the LLMs. All included studies used LLMs for data testing purposes only and were not used for real-time diagnosis of clinical patients. Table 1 shows the basic characteristics of the included studies.

**Table 1.** Characteristics and results of the eligible studies.

Study	Year	Country	Study type	Subjects	Case source	Sample size	LLM^a^	Comparison group	Outcome measures
Zhang et al [10]	2024	China	Prospective study	Ophthalmology cases	Patient visit records	26	GPT-4o	Ophthalmologists	c^b^, g^c^
Makhoul et al [11]	2024	Lebanon	Cross-sectional study	Otolaryngology cases	Published case reports	32	GPT-3.5	ENT^d^ physicians, FM^e^ specialists	a^f^, b^g^
Pillai et al [12]	2023	The United States	Cross-sectional study	Autoimmune diseases cases	Published case reports	40	GPT-3.5 GPT-4 LLaMa 2	A certified internist	a^f^, b^g^
Levin et al [13]	2024	Israel	Cross-sectional study	Neonatal cases	Developed by researchers	6	GPT-4 Claude-2.0	Certiﬁed neonatal nurse practitioners	c^b^, g^c^
Lyons et al [14]	2023	The United States	Cross-sectional study	Ophthalmology cases	Developed by researchers	44	GPT-4 Bing	Ophthalmology physicians	b^g^, d^h^
Sarangi et al [15]	2023	India	Cross-sectional study	General cases	Developed by researchers	120	GPT-3.5 Bard Bing	Radiology residents	a^f^
Paslı et al [16]	2024	Turkey	Prospective study	Emergency cases	Patient visit records	758	GPT-4	The ED^i^ triage team	d^h^
Wang et al [17]	2024	China	Retrospective cohort study	Thyroid cases	Patient visit records	109	GPT-4	Thyroid doctors	c^b^
Huang et al [18]	2024	The United States	Cross-sectional study	Ophthalmology cases	Patient visit records	20	GPT-4	Subspecialists (in glaucoma or retina)	c^b^, g^c^
Stoneham et al [19]	2023	UK	Retrospective study	Dermatology cases	Patient visit records	36	GPT-4	A dermatologist	a^f^
Hirosawa et al [20]	2023	Japan	Cross-sectional study	Internal medicine cases	Published case reports	52	GPT-3.5 GPT-4	GIM^j^ physicians	a^f^, b^g^
Horiuchi et al [21]	2025	Japan	Retrospective study	Musculoskeletal cases	Published case reports	106	GPT-4 GPT-4V	Radiologists	a^f^, b^g^
Mitsuyama et al [22]	2024	Japan	Retrospective study	Brain tumors cases	Patient visit records	150	GPT-4	Radiologists	a^f^, b^g^
Hirosawa et al [23]	2023	Japan	Retrospective cohort study	Internal medicine cases	Published case reports and developed by researchers	82	Bard	GIM^j^ physicians	a^f^, b^g^
Suh et al [24]	2024	Korea	Retrospective study	General cases	Published case reports	190	GPT-4V Gemini Pro Vision	Radiologists	b^g^
Fraser et al [25]	2023	The United States	Cross-sectional study	Emergency cases	Patient visit records	40	GPT-3.5 GPT-4	ED physician	a^f^, b^g^, d^h^
Hirosawa et al [26]	2023	Japan	Prospective study	Internal medicine cases	Developed by researchers	30	GPT-3.5	GIM^j^ physicians	a^f^, b^g^
Shemer et al [27]	2024	Israel	Retrospective cohort study	Ophthalmology cases	Patient visit records	63	GPT-3.5	Ophthalmology residents and ophthalmologists	a^f^, g^c^
Mohammadi et al [28]	2024	Iran	Retrospective study	Tibial plateau fracture cases	Retrospective study	111	GPT-4 GPT-4o	An ED physician and radiologist	f^k^
Arslan et al [29]	2025	Turkey	Prospective study	Emergency cases	Patient visit records	468	GPT-4 Copilot Pro	Triage nurses	d^h^
Rojas-Carabali et al [30]	2023	Singapore	Cross-sectional study	Ophthalmology cases	Developed by researchers	25	GPT-3.5 GPT-4	Ophthalmologists	a^f^, b^g^
Kaya et al [31]	2024	Germany	Retrospective study	Myocarditis cases	Patient visit records	396	GPT-4	Radiologists	a^f^, e^l^
Delsoz et al [32]	2024	The United States	Cross-sectional study	Ophthalmology cases	Published case reports	20	GPT-3.5 GPT-4	Cornea specialists	a^f^
Ming et al [33]	2024	China	Cross-sectional study	Ophthalmology cases	Published case reports	104	GPT-3.5 GPT-4	Ophthalmic residents	a^f^, b^g^
Nakaura et al [34]	2024	Japan	Retrospective study	Internal medicine cases	Patient visit records	28	GPT-2 GPT-3.5 GPT-4	Radiologists	a^f^, b^g^
Ito et al [35]	2023	Japan	Cross-sectional study	General cases	Published case reports	45	GPT-4	Emergency physicians	a^f^, d^h^
Gunes et al [36]	2024	Turkey	Cross-sectional study	thoracic cases	Published case reports	124	10 LLMs including GPT-3.5/4 Claude 3 Opus…	Published case reports	a^f^
Delsoz et al [37]	2023	The United States	Cross-sectional study	Ophthalmology cases	Published case reports	11	GPT-3.5	Ophthalmology residents	a^f^
Liu et al [38]	2023	China	Prospective study	Ophthalmology cases	Patient visit records	1226	GPT-3.5	Ophthalmologists	e^l^
Li et al [39]	2024	China	Retrospective study	Abdominal cases	Patient visit records	300	ERNie, 4.0 Claude 3.5 Sonnet	Radiologists	c^b^

a LLM: large language model.

b Accuracy score.

c Other auxiliary indicators (such as diagnostic completeness, diagnostic time, number of answers, etc).

d ENT: ear, nose, and throat.

e FM: family medicine.

f Frequency of correct primary diagnosis (answer).

g Frequency of correct diagnosis in a differential diagnosis list.

h Triage accuracy.

i ED: emergency department.

j GIM: general internal medicine.

k AUC: area under the curve.

l *F*_1_-score

### Quality of Evidence and Risk of Bias

The included articles were evaluated using the PROBAST tool, with the results presented in Multimedia Appendix 2. Overall, 10/30 (33.3%) studies had a low risk of bias, while 20/30 (66.6%) exhibited a high risk of bias. Regarding applicability, majority of study had low applicability concerns. Due to ethical concerns and patient privacy issues associated with the use of LLMs in clinical settings, most of the studies consist of retrospective studies with deidentified data and are limited to data testing. A total of 14 studies evaluated the diagnostic accuracy of models using small test sets. In addition, the “black box” nature of LLMs, whose training data are often undisclosed, complicates external evaluation and verification.

### LLM Feature Analysis

Although a total of 19 different LLMs were used in the included studies, extracting the LLM with the best diagnostic performance in studies tested with multiple large models simultaneously, we found that the optimal LLM did not belong to the GPT series in only 6 studies. In 80% (24/30) of the studies, the researchers chose to obtain and use the corresponding LLMs directly on the official website by online access, which somewhat lowered the threshold for the use of the LLMs in the medical field and made it more accessible to the public. In total, 18 of the included studies specified the date of access or version of the LLM used. Retrieval-augmented generation (RAG) is a technique that combines information retrieval and generation to enhance task performance by incorporating relevant information into LLMs [40]. RAG was mentioned in 2 of the studies by further training of pretrained models specific to task datasets, and although RAG has been widely used in large model studies, it needs to be strengthened in the medical field. Specific details can be found in Table 2.

**Table 2.** Characteristics of the large language models (LLMs) in eligible studies.

Study	Optimal LLM^a^ in research	Issuing company	Access mode	Date accessed (version)	Parameter settings	RAG^b^
Zhang et al [10]	GPT-4o	Open AI	—^c^	—	—	Unused
Makhoul et al [11]	GPT-3.5	—	Application-based ChatGPT 3.5	—	—	Unused
Pillai et al [12]	GPT-4	Open AI	Online access	August 12, 2023	—	Unused
Levin et al [13]	Claude-2.0	Anthropic	Platform developed by Anthropic (@Poe)	—	—	Unused
Lyons et al [14]	GPT-4	Open AI	Online access	March 19-24, 2023	—	Unused
Sarangi et al [15]	Bing	Microsoft	Search engine-based GPT-4	June 2023	—	Unused
Paslı et al [16]	GPT-4	Open AI	Online access	September 25, 2023	—	RAG
Wang et al [17]	GPT-4	—	Platform-based GPT-4 developed by researchers (ThyroAIGuide)	—	—	Unused
Huang et al [18]	GPT-4	Open AI	Online access	May 12, 2023	—	Unused
Stoneham et al [19]	GPT-4	Open AI	Online access	—	—	Unused
Hirosawa et al [20]	GPT-4	Open AI	Online access	April 10, 2023	—	Unused
Horiuchi et al [21]	GPT-4	Open AI	Online access	September 25, 2023	—	Unused
Mitsuyama et al [22]	GPT-4	Open AI	Online access	May 24, 2024	—	Unused
Hirosawa et al [23]	Bard	Google	Online access	June 8, 2023	—	Unused
Suh et al [24]	GPT-4V	Open AI	Online access	—	Temperature=1	Unused
Fraser et al [25]	GPT-3.5	Open AI	Online access	July 2023	—	Unused
Hirosawa et al [26]	GPT-3.5	Open AI	Online access	January 5, 2023	—	Unused
Shemer et al [27]	GPT-3.5	Open AI	Online access	March 2023	—	Unused
Mohammadi et al [28]	GPT-4o	Open AI	Online access	December 2023	—	Unused
Arslan et al [29]	GPT-4	Open AI	Online access	—	—	Unused
Rojas-Carabali et al [30]	GPT-4	Open AI	Online access	—	—	Unused
Kaya et al [31]	GPT-4	Open AI	Online access	March to July 2023	—	Unused
Delsoz et al [32]	GPT-4	Open AI	Online access	—	—	Unused
Ming et al [33]	GPT-4	Open AI	Online access	March 5-18, 2024	—	Unused
Nakaura et al [34]	Bing	Microsoft	Search engine-based GPT-4	—	—	Unused
Ito et al [35]	GPT-4	Open AI	Online access	March 15, 2023	—	Unused
Gunes et al [36]	Claude 3 Opus	Anthropic	Online access	May 2024	—	Unused
Delsoz et al [37]	GPT-3.5	Open AI	Online access	—	—	Unused
Liu et al [38]	GPT-3.5	Open AI	Online access	—	Temperature=0	Unused
Li et al [39]	Claude 3.5 Sonnet	Anthropic	Online access	June 13 to July 5, 2024	Temperature=1×10^-10^	RAG

a LLM: large language model.

b RAG: retrieval-augmented generation.

c Not available.

### Results of Diagnosis

The accuracy of the diagnoses made by the LLMs and the clinical professionals in the studies depends on the “standard answer” mentioned in the literature. The comparison is based on how their answers align with this standard. The “standard answer” in the included studies consists of the final diagnoses recorded in patient medical records or case reports, predetermined answers set by case developers, and diagnoses established by experienced clinical experts in the relevant departments.

### Application of LLMs in Clinical Diagnosis

The most common model task was the free text task, which appeared in 19 articles, while only 1 article involved a choice task. English was used for input and output in all but 2 articles: one used Hebrew for prompting, and the other used Chinese to compare model diagnostic performance. In LLM, prompt is an input mode that guides the model to specific tasks or generates specific outputs, typically including elements such as instructions (task descriptions), context (background information), examples, input data, output instructions, and roles [41,42]. When LLMs are used for case diagnosis, the most frequently used elements are commands and input data, which primarily include patient basic information, complaints, medical history, physical examination, and laboratory tests. The output content mainly consists of diagnostic lists or triage recommendations. The diagnostic accuracy of health care professionals in each study was evaluated by investigators or experts in relevant fields.

In studies where multiple LLMs were used to diagnose sample cases, only the data for the model with the best diagnostic performance were recorded. Of these studies, 85% (24/30) reported that the ChatGPT series models demonstrated the best diagnostic performance. Several investigators noted that the diagnostic accuracy of GPT models was comparable with that of physicians and did not show significant differences. Specific details can be found in Multimedia Appendix 3.

### Comparison of Diagnostic Accuracy Between LLMs and Health Care Professionals

Pooling the data revealed that 70% (21/30) of the studies used the frequency of correct diagnoses in model responses as the primary evaluation indicator of clinical diagnostic accuracy, excluding other auxiliary indicators. All accuracy results were expressed as percentages. For the optimal model, the accuracy of the primary diagnosis ranged from 25% to 97.8%, while triage accuracy ranged from 66.5% to 98%. In medical practice, the diagnostic agreement criterion is usually set at over 80%. The GPT series LLMs achieved diagnostic accuracy greater than 80% in clinical tasks across 3 studies in ophthalmology, 2 studies in general medicine, and 1 study each in radiology, emergency medicine, and general practice. Among the 7 studies focused on ophthalmic case diagnosis, the diagnostic performance was generally high, with 77.8% (7/9) of the large models showing diagnostic accuracy comparable with that of health care professionals.

In these cases, health care professionals received the same prompting words as the LLMs. In 60% (18/30) of the studies, control group participants were blinded to the true nature and goals of the study until it was completed. The diagnostic accuracy of health care professionals was compared with the outcomes of LLMs. The results showed that in 33.7% (20/30) of the studies, professionals had higher diagnostic accuracy than the models. In 33.3% (10/30) of the studies, the LLMs, specifically ChatGPT, had higher diagnostic accuracy than humans. The specific diagnostic accuracy comparisons are detailed in Table 3.

**Table 3.** Comparison of diagnostic accuracy between large language models (LLMs) and clinical professionals.

Specialty and study		Clinical professionals	Evaluation results (LLMs vs clinical professionals), %					
			a^a^	b^b^	c^c^	d^d^	e^e^	f^f^
Ophthalmology								
	Zhang et al [10]	3	—^g^	—	55 vs 74.7	—	—	—
	Lyons et al [14]	8	—	93 vs 95^h^	—	98.0 vs 86.0	—	—
	Huang et al [18]	15	—	—	50.4 vs 50.3	—	—	—
	Shemer et al [27]	6	68 vs 90	—	—	—	—	—
	Rojas-Carabali et al [30]	5	64 vs 85.6	72 vs 89.6^h^	—	—	—	—
	Delsoz et al [32]	3	85 vs 96.7	—	—	—	—	—
	Ming et al [33]	3	59.6 vs 60.6	76 vs 65.4^h^	—	—	—	—
	Delsoz et al [37]	3	72.7 vs 66.6	—	—	—	—	—
	Liu et al [38]	2	—	—	—	—	80.1 vs 89.4	—
Internal medicine								
	Hirosawa et al [20]	3	60 vs 50	81 vs 67^i^; 83 vs 75^j^	—	—	—	—
	Mitsuyama et al [22]	5	73 vs 69.4	94 vs 81.6^h^	—	—	—	—
	Hirosawa et al [23]	5	40.2 vs 64.6	53.7 vs 78^i^; 56.1 vs 82.9^j^	—	—	—	—
	Hirosawa et al [26]	2	53.3 vs 93.3	83.3 vs 98.3^i^	—	—	—	—
	Nakaura et al [34]	1	54 vs 100	96 vs 100^i^	—	—	—	—
	Li et al [39]	5	—	—	93.8 vs 99.6	—	—	—
Emergency department								
	Sinan Paslı et al [16]	Team	—	—	—	95.6 vs 92.8	—	—
	Fraser et al [25]	3	40 vs 47	63 vs 69^h^	—	—	—	—
	Arslan et al [29]	Team	—	—	—	66.5 vs 65.2	—	—
General medicine								
	Sarangi et al [15]	2	53.3 vs 60.4	—	—	—	—	—
	Suh et al [24]	8	—	48.9 vs 60.5^h^	—	—	—	—
	Ito et al [35]	3	97.8 vs 91.1	—	—	66.7 vs 66.7	—	—
Orthopedics								
	Horiuchi et al [21]	2	43 vs 47	58 vs 62.5^h^	—	—	—	—
	Mohammadi et al [28]	2	—	—	—	—	—	0.73 vs 0.74
Cardiothoracic								
	Kaya et al [31]	3	81 vs 91.3	—	—	—	85 vs 92.7	—
	Gunes et al [36]	2	70.3 vs 46.8	—	—	—	—	—
Otolaryngology								
	Makhoul et al [11]	20	—	70.8 vs 71.3^h^	—	—	—	—
Immunology								
	Pillai et al [12]	1	25 vs 47.5	45 vs 60^i^; 47.5 vs 75^j^	—	—	—	—
Neonatology								
	Levin et al [13]	32	—	—	70.8 vs 82.5	—	—	—
Thyroid								
	Wang et al [17]	40	—	—	73.6 vs 87.4	—	—	—
Dermatology								
	Stoneham et al [19]	1	56 vs 83	—	—	—	—	—

a Frequency of correct primary diagnosis (answer)

b Frequency of correct diagnosis in the 3, 5, or 10 differential diagnoses.

c Accuracy score

d Triage accuracy

e *F*_1_-score.

f AUC: area under the curve.

g Not available.

h Frequency of correct diagnosis in the 3 differential diagnoses.

i Frequency of correct diagnosis in the 5 differential diagnoses.

j Frequency of correct diagnosis in the 10 differential diagnoses.

### Meta-Analysis

**Figure 2.**
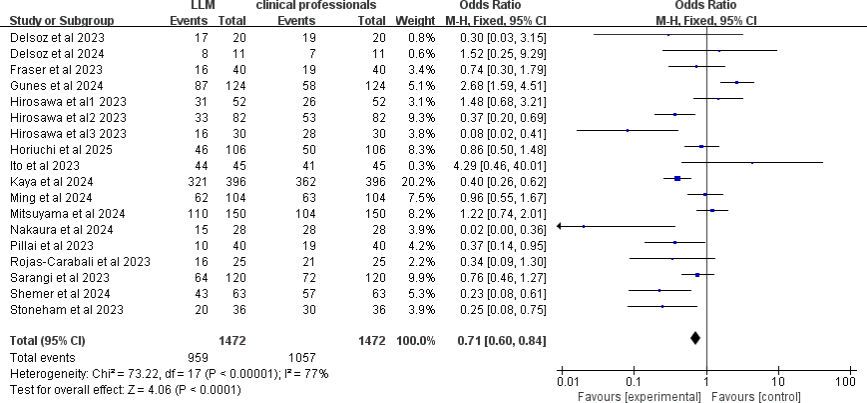
Forest plot comparing diagnostic accuracy of large language models (LLMs) and clinical professionals [37,32,25,36,20,23,26,21,35,31,33,22,34,12,30,15,27,19].

Although this paper synthesizes over 4000 clinical cases, these cases exhibit significant heterogeneity in terms of clinical departments, diagnostic methodologies, and evaluation metrics. Due to these inherent differences, only 18 studies that used primary diagnostic accuracy as the evaluation metric were included in a meta-analysis. The analysis revealed that clinical professionals generally outperformed LLMs in diagnostic accuracy across various conditions, as shown in Figure 2. The *P* value was less than 0.05, and the *I*² value was 77%, indicating significant heterogeneity among the studies. Sensitivity analysis did not significantly improve the heterogeneity. Subgroup analyses by clinical department showed reduced heterogeneity in ophthalmology-related research, yet results still favored the diagnostic accuracy of ophthalmology professionals over LLMs.

## Discussion

### Principal Findings

In this systematic review, we analyzed the diagnostic accuracy of LLMs compared with clinical professionals, encompassing various LLMs and common medical specialties. Although the results typically indicated superior diagnostic accuracy among professionals, this study compiled the methodologies, functionalities, and outcomes of using LLMs in medical diagnostics. It affirmed the diagnostic capabilities of generic LLMs, providing evidence for their potential as healthcare assistants.

### Application of LLMs in Clinical Diagnosis Still in Exploratory Stage

This review includes only peer-reviewed and published literature, so the models examined in the included studies primarily use text-based input and output for diagnostic tasks. However, with the advancement of large models, multimodal capabilities have also been integrated [43]. Some preprint studies [44,45] have explored using GPT-4V, incorporating imaging data into input prompts. Notably, adding images to LLM did not improve diagnostic performance. In a study by Horiuchi et al [44], ChatGPT-4, which relied solely on text prompts, achieved higher diagnostic accuracy compared to GPT-4V, which combined text and images. Without few-shot learning, LLMs may struggle with image recognition and interpretation, sometimes leading to counterproductive outcomes.

Currently, the performance of general LLMs continues to improve, showing strong results in health care question answering, text classification, and clinical concept extraction [46]. However, these studies remain experimental and laboratory-based. Issues such as the interpretability of model responses and medical ethics pose significant challenges to applying these models in real clinical settings. Furthermore, the trust and acceptance of AI models by clinicians directly affect their adoption and implementation. Therefore, education and training programs are crucial for enhancing physicians’ AI literacy [47].

### Evolution of Artificial Intelligence in Clinical Diagnosis

The evolution of AI in clinical diagnosis has progressed from simple specialized systems to complex deep learning models. Early AI systems were based on fixed rules and expert knowledge bases. While these systems achieved some success in specific tasks, they had limited scalability and flexibility. The advancement of deep learning technologies, particularly the emergence of LLMs, has ushered AI applications in the health care sector into a new era [48,49].

LLM can learn from vast amounts of medical data to autonomously discover and summarize diagnostic rules, significantly enhancing diagnostic accuracy and reliability. The development of RAG technology and fine-tuning techniques has further enabled LLM to acquire advanced domain expertise and effectively perform specialized tasks.

### Ethics of Artificial Intelligence in Clinical Diagnosis

Although the pace of artificial intelligence development is swift, its broad implementation in clinical settings continues to encounter numerous obstacles, including concerns over data privacy, accountability, and ethics. Consequently, numerous scholars [50-53] underscore the imperative of utmost caution in using these technologies. Advances in the future will necessitate not only technological innovations but also comprehensive enhancements in legal and ethical frameworks to ensure that AI technology is safely and effectively woven into clinical diagnostic processes. In deploying LLMs within actual clinical workflows, it is crucial to first guarantee the transparency of all used data and secure patients’ informed consent. In addition, to tackle potential biases within AI models, periodic audits are advised to identify and amend any discrepancies. Furthermore, to safeguard patient safety and adhere to regulatory demands, medical institutions should work alongside legal and ethical experts to establish stringent guidelines and oversight mechanisms for AI use. For instance, forming an ethics committee to assess and monitor AI applications could ensure compliance with ethical standards and legal requirements. These targeted measures are essential to surmount existing challenges and foster the successful incorporation of AI technologies in clinical diagnostics.

### Application of LLMs in Specific Medical Fields

The application of LLMs in the medical field is gradually expanding, especially in imaging diagnosis, clinical decision support, and personalized treatment planning. Due to their specific needs and challenges, each medical field shows different ways and effects of LLMs’ application.

Ophthalmology is one of the pioneers of LLMs’ applications. In ophthalmic diagnosis, imaging data such as fundus images, retinal scans are typically complex, but LLMs excel in processing and analyzing these types of data [45,54]. Research has shown that LLMs can identify minor lesions in fundus images and diagnose conditions such as glaucoma and macular degeneration [55,56]. Ophthalmic diagnostics rely not only on imaging but also on additional data such as patients’ genetic information and blood sugar levels. In the future, LLMs could integrate these multimodal data to achieve more accurate disease predictions through personalized treatment. Particularly in resource-limited areas, easily accessible LLMs with low usage thresholds could replace some ophthalmologists in preliminary screenings, further providing efficient diagnostic support in remote regions.

There are many internal medicine diseases that require long-term follow-up and monitoring. LLMs can process all historical data of patients simultaneously and updating personalized treatment plans, assisting clinical professionals in making more beneficial decisions [57-59]. In the future, LLMs will be paired with wearable devices to monitor patients’ health in real time, predict potential risks through data analysis, and provide early intervention for patients with medical diseases, thereby reducing the incidence and recurrence of the disease.

In the fields of otolaryngology [60,61] and dermatology [62,63], LLMs have been used to analyze imaging data for detecting lesions in respective areas. The latest models now offer voice input features, allowing patients to use the model anytime and anywhere to help in the early detection of speech disorders and vocal cord issues. In the future, integrating voice recognition with physiological data can also assist physicians in more accurately locating lesion areas during otolaryngological surgeries, thus improving treatment efficacy. Furthermore, by combining images of skin lesions with patients’ genetic data, LLMs can help predict the risk of dermatological conditions and provide early warnings.

### Exploration of the Use of LLMs in Various Clinical Departments

Currently, extensive research in fields such as ophthalmology, internal medicine, and radiology has demonstrated the substantial potential of LLMs in clinical diagnostics and pathological analysis. These models have even been implemented in some hospitals. Many clinical professionals are actively exploring how to integrate these technologies into their daily diagnostic and treatment routines.

However, the application of LLMs in other specialized areas remains limited, and research in these fields appears to be lacking. Several reasons account for this disparity: First, the departments mentioned above primarily focus on diagnostic issues, providing rich training data for large models, especially in terms of imaging and case data. Second, the main challenges these departments face in clinical practice, such as accurate diagnosis and disease prediction, are areas where LLMs can excel. In contrast, other departments such as surgery, although also using imaging data, face complexities in surgical and procedural tasks that hinder the maturity of AI applications. Gynecology has seen some applications of image recognition, but lacks depth in research and sufficient data accumulation, making model training challenging. In addition, real-world factors such as data privacy protection and technology dissemination also restrict the application of large models in certain departments.

### Future Directions

The “human-AI collaboration” model involves an initial diagnosis provided by AI, which is then reviewed and confirmed by clinicians. AI’s capability to analyze clinical data in real time enables it to offer personalized monitoring plans based on the specific conditions of patients. This continuous tracking of patient health and treatment outcomes helps achieve the goals of personalized medicine and precise diagnosis [64,65]. In addition, AI can provide customized services and recommendations based on user preferences and backgrounds, enhancing user experience and effectiveness. This model combines the rapid processing capabilities of AI with the expert judgment of clinicians, improving the efficiency and reliability of clinical trials. It also enhances data analysis and patient management, offering significant advantages in cost reduction, resource use, and ensuring the reliability of trial results.

Although LLMs are not inherently designed for clinical diagnostic tasks, advancements in technology and data accumulation are expected to improve their performance in clinical settings. Techniques such as large-scale medical literature analysis, specific clinical data training, task-specific fine-tuning, personalized training for particular scenarios, and integration with APIs or other supplementary software tools are anticipated to enhance the diagnostic support and treatment recommendations provided by these models [66,67]. Hybrid models could be developed by combining rule-based clinical decision support systems with the pattern recognition capabilities of LLMs. For example, Vision China 2023 introduced Eye GPT [68], a system that integrates ophthalmic medical knowledge with LLM. This system aims to assist clinicians in disease diagnosis and improve medical efficiency by combining extensive ophthalmic information with powerful computational capabilities. This innovation in integrating large models with specialized clinical fields is expected to play a crucial role in future clinical applications and provide research directions for other medical specialties.

### Limitations

This study has several limitations. First, the inclusion criteria restricted the review to studies comparing the diagnostic accuracy of LLMs with that of clinical health care professionals using case groups. This limitation may affect the comprehensiveness of the review and introduce selection bias. In addition, there is no specialized tool for assessing the risk of bias in literature related to LLMs. Although PROBAST was used to evaluate the quality of the included studies, its focus on diagnostic accuracy may influence the evaluation results. Finally, significant heterogeneity among the studies was observed, with variations in outcome measures potentially related to differences in intervention subjects, prompt inputs, and information modalities. Further exploration of LLM diagnostic performance is needed through large-scale, multicenter, and high-quality cross-sectional and cohort studies.

### Conclusions

This systematic review included 20 studies comparing the diagnostic accuracy of LLMs with that of health care professionals, encompassing various generative AI models and medical specialties. The findings indicate that while LLMs still have a long way to go in accurately diagnosing real-world clinical scenarios and currently lack the level of understanding of human experts, they undeniably possess significant potential as health care assistants. With ongoing advancements and optimizations in technology, it is anticipated that LLMs will play an increasingly important role in future clinical diagnostics.

## Supplementary material

10.2196/64963 Multimedia Appendix 1 Details of the search strategy in PubMed.

10.2196/64963 Multimedia Appendix 2 Quality assessment of included studies.

10.2196/64963 Multimedia Appendix 3 Characteristics of large language models (LLMs) applied in clinical diagnostic studies.

10.2196/64963 Checklist 1 PRISMA (Preferred Reporting Items for Systematic Reviews and Meta-Analysis) checklist.

## Abbreviations

AI: artificial intelligence
CINAHL: Cumulative Index to Nursing and Allied Health Literature
CNKI: China National Knowledge Infrastructure
LLM: large language model
MeSH: Medical Subject Heading
PRISMA-DTA: Preferred Reporting Items for Systematic Reviews and Meta-analysis of Diagnostic Test Accuracy Studies
PROBAST: Prediction Model Risk of Bias Assessment Tool
RAG: retrieval-augmented generation
