# Supplementary material for: Comparing Diagnostic Accuracy of Clinical Professionals and Large Language Models: Systematic Review and Meta-Analysis
Source: JMIR Med Inform. 2025 Apr 25;13:e64963. doi: 10.2196/64963 (PMC12047852; doi:10.2196/64963)
Supplement: Multimedia Appendix 1 [file medinform-v13-e64963-s001.docx]

**Multimedia Appendix 1: Details of the Search Strategy and Study Selection**

Takes PubMed as an example, and the search strategy is as follows:

#1 (((((((((((((((((("large language model"[Title/Abstract]) OR ("LLM"[Title/Abstract])) OR ("generative artificial intelligence"[Title/Abstract])) OR ("generative AI"[Title/Abstract])) OR ("generative pretrained transformer"[Title/Abstract])) OR ("dialogue generation"[Title/Abstract])) OR ("Bard"[Title/Abstract])) OR ("BERT"[Title/Abstract])) OR ("Bing"[Title/Abstract])) OR ("GPT"[Title/Abstract])) OR ("PaLM"[Title/Abstract])) OR ("CLIP"[Title/Abstract])) OR ("DALL-E"[Title/Abstract])) OR ("Llama"[Title/Abstract])) OR ("Sora"[Title/Abstract])) OR ("GLM"[Title/Abstract])) OR ("Qwen"[Title/Abstract])) OR ("ERNIE"[Title/Abstract]))

#2 (((((((Diagnosis[MeSH Terms]) OR (Clinical Reasoning[MeSH Terms])) OR (Diagnos*[Title/Abstract])) OR (Reasoning, Clinical[Title/Abstract])) OR (Clinical Judgement[Title/Abstract])) OR (identification[Title/Abstract])) OR (assessment[Title/Abstract]))

#3 (((((((((Data Accuracy[MeSH Terms]) OR (Outcome and Process Assessment, Health Care[MeSH Terms])) OR (Symptom Assessment[MeSH Terms])) OR (Accuracy, Data[Title/Abstract])) OR (Data Quality[Title/Abstract])) OR (Accuracy[Title/Abstract])) OR (Correct* Rate[Title/Abstract])) OR (Outcome and Process Assessment[Title/Abstract])) OR (correctness[Title/Abstract]))

#4 (((((((("Cross-Sectional Studies"[Mesh]) OR ("Cohort Studies"[Mesh])) OR ("Prospective Studies"[Mesh])) OR ("Retrospective Studies"[Mesh])) OR (retrospective study)) OR (cross-sectional study)) OR (cohort study)) OR (Prospective study))

#5 ("2017/01/01"[Date - Publication] : "3000"[Date - Publication])

#6 ((("Medicine"[Mesh]) OR (clinical[Title/Abstract])) OR (medicine[Title/Abstract])) OR (medical[Title/Abstract])

#7 Filters: Humans, English

#8 #1 AND #2 AND #3 AND #4 AND #5 AND #6 AND #7
