# Supplementary material for: Comparing Diagnostic Accuracy of Clinical Professionals and Large Language Models: Systematic Review and Meta-Analysis
Source: JMIR Med Inform. 2025 Apr 25;13:e64963. doi: 10.2196/64963 (PMC12047852; doi:10.2196/64963)
Supplement: Multimedia Appendix 2 [file medinform-v13-e64963-s002.docx]

**Multimedia Appendix 2: Quality assessment of included studies**

| **Study** | **Risk of bias** | | | | **Applicability** | | |
| --- | --- | --- | --- | --- | --- | --- | --- |
|  | **participants** | **outcome** | **analysis** | **overall** | **participants** | **outcome** | **overall** |
| Junxiu Zhang et al | Low | Low | Low | Low | Low | Low | Low |
| Mikhael Makhoul et al | Low | Low | Low | Low | Low | Low | Low |
| Joshua Pillai et al | Low | High | Low | High | Low | Low | Low |
| Chedva Levin et al | Low | Low | High | High | Low | Low | Low |
| Riley J. Lyons et al | Low | High | Low | High | Low | Low | Low |
| Pradosh Kumar Sarangi et al | Low | Low | Low | Low | Low | Low | Low |
| Sinan Paslı et al | Low | Low | Low | Low | Low | Low | Low |
| Zhixiang Wang et al | High | Low | Low | High | Low | Low | Low |
| Andy S. Huang et al | Low | Low | Low | Low | Low | Low | Low |
| Sophie Stoneham et al | High | Low | Low | High | Low | Low | Low |
| Takanobu Hirosawa et al | Low | High | Low | High | Low | Low | Low |
| Daisuke Horiuchi et al | High | Low | Low | High | Low | High | High |
| Yasuhito Mitsuyama et al | High | Low | Low | High | Low | Low | Low |
| Takanobu Hirosawa et al | High | Low | Low | High | Low | Low | Low |
| Pae Sun Suh et al | High | Low | Low | High | Low | Low | Low |
| Hamish Fraser et al | Low | Low | High | High | Low | Low | Low |
| Takanobu Hirosawa et al | Low | High | Low | High | Low | Low | Low |
| Asaf Shemer et al | High | Low | Low | High | Low | Low | Low |
| Mohammadreza Mohammadi et al | High | Low | Low | High | Low | Low | Low |
| Banu Arslan et al | Low | Low | Low | Low | Low | Low | Low |
| William Rojas-Carabali et al | Low | Low | High | High | Low | Low | Low |
| Kenan Kaya et al | High | Low | High | High | Low | Low | Low |
| Mohammad Delsoz et al | Low | Low | Low | Low | Low | Low | Low |
| Shuai Ming et al | Low | Low | Low | Low | Low | Low | Low |
| Takeshi Nakaura et al | Low | High | Low | High | Low | Low | Low |
| Naoki Ito et al | Low | High | Low | High | Low | Low | Low |
| Yasin Celal Gunes et al | Low | Low | Low | Low | Low | Low | Low |
| Mohammad Delsoz et al | Low | Low | High | High | Low | Low | Low |
| Xiaocong Liu et al | Low | Low | Low | Low | Low | Low | Low |
| Chao Li et al | High | Low | Low | High | Low | Low | Low |
