# Supplementary material for: Comparing Diagnostic Accuracy of Clinical Professionals and Large Language Models: Systematic Review and Meta-Analysis
Source: JMIR Med Inform. 2025 Apr 25;13:e64963. doi: 10.2196/64963 (PMC12047852; doi:10.2196/64963)
Supplement: Multimedia Appendix 3 [file medinform-v13-e64963-s003.docx]

**Multimedia Appendix 3: Characteristics of LLMs applied in clinical diagnostic studies**

| **Study** | **Modality** | **Language** | **Model task** | **Prompt** | **Input** | **Output** | **Evaluation mode** | **Conclusion** |
| --- | --- | --- | --- | --- | --- | --- | --- | --- |
| Junxiu Zhang et al | Text-Text | English | Free text | a, b, e, f | g, h, i, j | A primary diagnosis and a differential diagnosis list | The independent ophthalmologists assessed accuracy and completeness using Likert scales. | AI, including GPT-4o, is currently not an acceptable standalone method for diagnosing glaucoma due to its lower accuracy compared to human clinicians. |
| Mikhael Makhoul et al | Text-Text | English | Free text | / | g, h, k | Diagnostic results, differential diagnosis | Three researchers evaluated the results based on the correct diagnosis established by the principal investigator. | GPT-3.5 exhibited high accuracy in diagnosing real-life ENT case scenarios, matching the accuracy rates of ENT physicians, thereby surpassing FM specialists. |
| Joshua Pillai et al | Text-Text | English | Free text | b, e, f | g, h, j, k | Ten possible diagnoses | The researchers evaluated the results based on the case report. | It was found that GPT-4 did not significantly differ in correctly identifying DIRA and FMF patients compared to the internist. |
| Chedva Levin et al | Text-Text | Hebrew | Free text | / | g, h, j, k | Diagnostic results, management plan | Two expert nurses evaluated the clinical decision-making performance for each case scenario using a 100-point scale. | There is a discrepancy in accuracy between the clinical reasoning diagnoses of Claude-2.0 and those of neonatal nurses. |
| Riley J. Lyons et al | Text-Text | English | Free text | a, e, (b, f) | g, h, k | Three possible diagnoses, triage recommendation | Two researchers independently scored the responses based on the answers provided for the virtual cases. | GPT-4 offered high diagnostic and triage accuracy that was comparable to the physician respondents. |
| Pradosh Kumar Sarangi et al | Text-Text | English | Choice | / | g, l | Choice answer | The researchers evaluated the responses based on the answers to the developed case questions. | The residents consistently outperformed the AI models in terms of accuracy. |
| Sinan Paslı et al | Text-Text | English | Free text | b, c, e, f | g, h, k | Triage recommendation | The researchers evaluated the results based on the patient triage areas determined by emergency medicine experts. | GPT-4 possess outstanding predictive skills in triaging patients in an emergency setting. |
| Zhixiang Wang et al | Text-Text | English | Free text | b, e, f | j | Diagnosis (TI-RADS classification), treatment recommendation | The researchers collected and analyzed the report scores, requiring physicians to read the reports and rate them on a scale of 1 to 5. | There are differences in diagnostic accuracy between GPT-4 and physicians, indicating a need for further improvement. |
| Andy S. Huang et al | Text-Text | English | Free text | a, b, c, e, f | g, h, j, k | Diagnostic results, management plan | The researchers used a 10-point Likert scale to measure accuracy based on the case reports. | The LLM chatbot outperformed glaucoma specialists and matched retina specialists in diagnostic and treatment accuracy |
| Sophie Stoneham et al | Text-Text | English | Free text | / | g, h, j, k | Diagnostic results | The researchers evaluated the results based on the case report. | Although GPT-4 demonstrates diagnostic capabilities, it currently differs from dermatologists in terms of accuracy. |
| Takanobu Hirosawa et al | Text-Text | English | Free text | b, e | g, h, j, k | Ten possible diagnoses | The researchers evaluated the results based on the case report. | With a diagnostic accuracy rate exceeding 80%, GPT-4 exhibits diagnostic capabilities comparable to those of physicians. |
| Daisuke Horiuchi et al | Text(+Image)-Text | English | Free text | a, b, e, f | g, h, l | Three possible diagnoses | Two researchers evaluated diagnoses whether they were consistent with the actual ground truth in consensus. | While GPT-4-based ChatGPT’s diagnostic performance was comparable to radiology residents, it did not reach the performance level of board-certified radiologists in musculoskeletal radiology. |
| Yasuhito Mitsuyama et al | Text-Text | English | Free text | b, e | l | Three possible diagnoses | The researchers evaluated the results based on the pathological diagnosis of the tumor. | GPT-4 exhibited good diagnostic capability, comparable to neuroradiologists in differentiating brain tumors from MRI reports. |
| Takanobu Hirosawa et al | Text-Text | English | Free text | b, e | g, h, j, k | Ten possible diagnoses | One researcher evaluated the results based on the case reports and the simulated case answers. | Although there are differences in diagnostic accuracy between GPT-4 and physicians, there is potential for improvement and refinement. |
| Pae Sun Suh et al | Text(+Image)-Text | English | Free text | b, c, e, f | h, l | Three possible diagnoses | The researchers evaluated the results based on the case report. | Although GPT-4V slightly underperformed compared with radiologists, it nonetheless demonstrated promising potential as a supportive tool in diagnostic decision-making. |
| Hamish Fraser et al | Text-Text | English | Free text | / | g, h, k | Three possible diagnoses, triage recommendation | The researchers evaluated the results based on the patient’s medical records. | In emergency department cases, the diagnostic accuracy of GPT-3.5 is lower than that of the control group of physicians. |
| Takanobu Hirosawa et al | Text-Text | English | Free text | b, e | g, h, j, k | Ten possible diagnoses | The researchers evaluated the diagnoses based on the clinical case answers generated by the three authors. | The diagnostic lists generated by GPT-3.5 exhibit high accuracy for clinical cases with common chief complaints. However, there are still differences compared to physicians. |
| Asaf Shemer et al | Text-Text | English | Free text | b, e | g, h, k, (j) | Three possible diagnoses | The researchers evaluated the results based on the patient’s medical records. | In the field of ophthalmology, GPT-3.5's overall diagnostic capability is significantly inferior compared to that of resident ophthalmologists and experienced ophthalmologists. |
| Mohammadreza Mohammadi et al | Text(+Image)-Text | English | Judgment Free text | b, e, f | l | Fracture diagnosis | The researchers evaluated the results based on the patient’s medical records. | ChatGPT-4o matched the physicians’ performance and also had the highest specificity. |
| Banu Arslan et al | Text-Text | English | Free text | b, e | g, h, i, j, k | Triage recommendation | The EM physician determined the appropriate triage level and evaluated the results. | ChatGPT and Copilot outperform traditional nurse triage in identifying high-acuity patients. |
| William Rojas-Carabali et al | Text-Text | English | Free text | / | g, h, j, k | Three possible diagnoses | The researchers evaluate the responses based on the virtual case answers. | In ophthalmology cases, the diagnostic accuracy of GPT-4 is lower than that of the control group of physicians. |
| Kenan Kaya et al | Text-Text | English | Judgment | b, e, f | g, j | Yes or no | The reference standard was established by the diagnosis of myocarditis based on the assessment of two radiologists. | GPT-4 yielded good accuracy for diagnosing myocarditis based on CMR reports in a large dataset from multiple centers. |
| Mohammad Delsoz et al | Text-Text | English | Free text | b, e | g, h, j, k | Diagnostic results | The researchers evaluated the results based on the case report. | In the diagnosis of corneal eye diseases, the diagnostic accuracy of GPT-4 is comparable to that of the control group of physicians, with no statistically significant difference. |
| Shuai Ming et al | Text-Text | English | Free text | a, b, e, f | g, h, j, k | Three possible diagnoses | The researchers evaluated the results based on the case report. | While GPT-3.5 underperformed, GPT-4.0 approached and numerically surpassed residents in differential diagnosis. |
| Takeshi Nakaura et al | Text-Text | English | Free text | a, b, c, e, f | g, h, j, k | Most five possible diagnoses | The researchers evaluated the results based on the patient’s medical records. | In radiology reports, GPT-4's primary diagnostic accuracy differs from that of the control group of physicians, although there is no significant difference in the top five differential diagnoses. |
| Naoki Ito et al | Text-Text | English | Free text | b, c, e, f | g, h, j, k | Diagnostic results, triage recommendation | Two researchers independently evaluated the results based on the case reports. In case of discrepancies. | In general practice cases, GPT-4's preliminary diagnostic accuracy is superior to that of the control group of physicians, while its triage accuracy is comparable to that of the control group. |
| Yasin Celal Gunes et al | Text-Text | English | Free text | a, b, e, f | g, h, l | Five possible diagnoses | The researchers evaluated the results based on the case report. | Claude 3 Opus outperformed other LLMs and radiologists in text-based thoracic radiology cases. |
| Mohammad Delsoz et al | Text-Text | English | Free text | b, e | g, h, j, k | Diagnostic results | The researchers evaluated the results based on the case report. | In glaucoma cases, the diagnostic accuracy of GPT-3.5 is comparable to or exceeds that of senior ophthalmology residents. |
| Xiaocong Liu et al | Text-Text | Chinese / English | Free text | b, c, e, f | j | Diagnostic results | The researchers evaluated the results based on the case report. | GPT-3.5 can serve as a useful medical assistant, but there remains a gap in diagnostic accuracy compared to professionals. |
| Chao Li et al | Text-Text | Chinese | Free text | / | j | Diagnostic reports | The researchers assessed accuracy and completeness using Likert scales. | Claude 3.5 Sonnet yields comparable performance to radiologists in generating radiology reports. |

**Prompt words: ^a.^** Role Play: This involves instructing the model to generate responses or perform tasks based on a specified role or identity. For example, you might ask the model to respond as if it were a medical expert, a historian, or a fictional character. **^b.^** Instruction: This directs the model to perform a specific task or follow a set of instructions to achieve a desired outcome. It tells the model what type of task is needed, such as summarizing a text, generating a report, or answering a question. **^c.^** Contextual Information: This provides background or additional information that helps the model better understand the task. Contextual information includes relevant details that set the stage for the model’s response, such as historical background, situational details, or specific conditions. **^d.^** Example: This gives the model a concrete demonstration of what is required. By providing examples, you show the model how to perform the task or what the expected output should look like. Examples help clarify the format and content of the desired response. **^e.^** Input Data: This refers to the specific information or data that the model is expected to process or analyze. Input data can be text, numbers, or any other relevant information that the model needs to use to generate a response. **^f.^** Output Instructions: These specify the type or format of the response that the model should produce. Output instructions guide how the results should be structured, such as whether the response should be in bullet points, a summary, or a detailed report.

**Input Content:** **^g.^** Patient profile, **^h^**^.^ Medical history, **^i.^** Symptoms, **^j.^** Examination findings, **^k.^** Complaints, **^l.^** medical images or image description
